# The implementation of the Medical Regulation Office and Mobile Emergency Attendance System and its impact on the gravity profile of non-traumatic afflictions treated in a University Hospital: a research study

**DOI:** 10.1186/1472-6963-7-173

**Published:** 2007-10-24

**Authors:** Sérgio LB Lopes, José Sebastião dos Santos, Sandro Scarpelini

**Affiliations:** 1 Department of Medicine, University Hospital, Faculty of Medicine of Ribeirão Preto, University of São Paulo. Rua Bernardino de Campos, 1000. Ribeirão Preto. São Paulo. Brazil; 2 Department of Surgery and Anatomy, University Hospital of the University of São Paulo School of Medicine at Ribeirão Preto. Av. Bandeirantes, 3900. Ribeirão Preto. São Paulo. Brazil; 3 Department of Surgery and Anatomy, University Hospital of the University of São Paulo School of Medicine at Ribeirão Preto. Rua Bernardino de Campos, 1000. Ribeirão Preto. São Paulo. Brazil

## Abstract

**Background:**

The public health system of Brazil is structured by a network of increasing complexity, but the low resolution of emergency care at pre-hospital units and the lack of organization of patient flow overloaded the hospitals, mainly the ones of higher complexity. The knowledge of this phenomenon induced Ribeirão Preto to implement the Medical Regulation Office and the Mobile Emergency Attendance System. The objective of this study was to analyze the impact of these services on the gravity profile of non-traumatic afflictions in a University Hospital.

**Methods:**

The study conducted a retrospective analysis of the medical records of 906 patients older than 13 years of age who entered the Emergency Care Unit of the Hospital of the University of São Paulo School of Medicine at Ribeirão Preto. All presented acute non-traumatic afflictions and were admitted to the Internal Medicine, Surgery or Neurology Departments during two study periods: May 1996 (prior to) and May 2001 (after the implementation of the Medical Regulation Office and Mobile Emergency Attendance System). Demographics and mortality risk levels calculated by Acute Physiology and Chronic Health Evaluation II (APACHE II) were determined.

**Results:**

From 1996 to 2001, the mean age increased from 49 ± 0.9 to 52 ± 0.9 (P = 0.021), as did the percentage of co-morbidities, from 66.6 to 77.0 (P = 0.0001), the number of in-hospital complications from 260 to 284 (P = 0.0001), the mean calculated APACHE II mortality risk increased from 12.0 ± 0.5 to 14.8 ± 0.6 (P = 0.0008) and mortality rate from 6.1 to 12.2 (P = 0.002). The differences were more significant for patients admitted to the Internal Medicine Department.

**Conclusion:**

The implementation of the Medical Regulation and Mobile Emergency Attendance System contributed to directing patients with higher gravity scores to the Emergency Care Unit, demonstrating the potential of these services for hierarchical structuring of pre-hospital networks and referrals.

## Background

The Unified Health System of Brazil, instituted in 1988, is organized into a network of increasing complexity, with integrated, regionalized hierarchies that ensure that the principles of equity in access to health resources, universality and integration of care are guaranteed for all citizens [1-3]. However, these legal measures have failed to provide solutions for daily hardships, especially within the emergency care system [4,5].

With the clear expansion of primary care and pre-hospital emergency care, which has occurred with international financial help, the general clinical attendance and emergency care management continue to be centralized in hospitals designed to guarantee first aid to patients presenting acute diseases of all levels of severity, creating excessive demands on hospitals of greater complexity located in large urban centers such as the city of Ribeirão Preto [4].

Ribeirão Preto is a city of 504,923 inhabitants, 72% of them depend on public health care [6]. The primary care network is structured into 32 basic units and 5 family health units. The city counts, too, with 13 specialized ambulatory units, 5 of them designed for emergency care. The hospital network consists of 4 general hospitals and 1 obstetric hospital, with a total of 1115 beds offered to the public health system. Due to its affiliation with the University of São Paulo School of Medicine at Ribeirão Preto, the University Hospital is a reference center for a population of more than five million from five districts of São Paulo [6]. It has 761 beds distributed between the University Campus Unit (with 604 beds for elective admissions) and the Emergency Center (EC) downtown (with 157 beds for emergency admissions, being 43 for critical care [4]).

The Mobile Emergency Attendance System (SAMU), which was instituted in 1996, includes one advanced mobile emergency unit, staffed by doctors and nurses, and 12 basic mobile emergency units, staffed by professionals trained in basic life support. In an attempt to organize the health services in a hierarchical manner and to guarantee the management of patient flow within the emergency care system, the Medical Regulation Office (MRO) was instituted in 2000, in a process which considered the experiences of similar systems in place in North America and Europe [7-12]. The office began to regulate the flow of emergency cases to the hospitals, with popular support, respecting the hierarchical position of each institution and the actual level of care available at the various health care centers.

From this perspective, the objective of this study was to analyze the impact of these services on the gravity profile of non-traumatic affections at the EC.

## Methods

The study, conducted after being approved by our Ethics committee, involved 906 patients older than 13 years of age (patients younger than that age were generally cared for by pediatricians) who presented non-traumatic afflictions and were admitted to the EC in the areas of Internal Medicine, Surgery and Neurology during two different one-month periods: May 1996 (474 patients) and May 2001 (432 patients). The first study period (May 1996) was chosen because it preceded the creation of the SAMU and its MRO. At the time of the second study period (May 2001), the SAMU and MRO had been fully operational for at least one year.

The populations studied during the two cited periods were analyzed in terms of general demographic data, origin, presence or absence of other medical affections besides the principal diagnosis (co-morbidities) and in-hospital complications (any secondary diagnosis that appeared during the hospital stay, reflecting an organic affliction other than the principal diagnosis).

**Figure 1:**
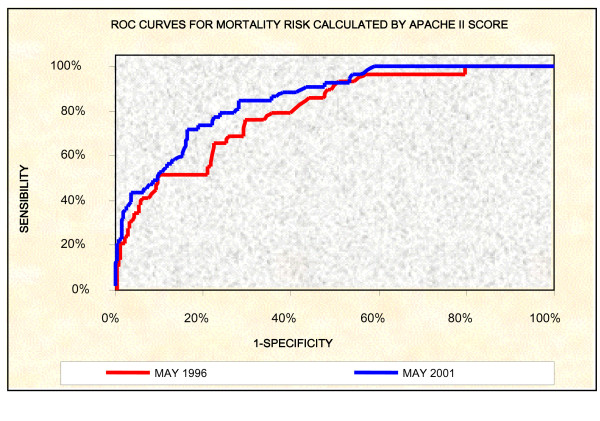
Roc curves for mortality risk calculated by APACHE II score.

Data were collected from medical records of patients admitted during the study periods, following a survey performed in the hospital data processing center. The mortality risk for each patient was calculated during the first 24 hours of hospitalization using the Acute Physiology and Chronic Health Evaluation II (APACHE II) score, which was paired with the observed mortality for the two periods of study using the ROC curve analysis, with an area under the curve of 0.801 (95% CI 0.723 to 0.878) and 0.855 (95% CI 0.805 to 0.904), respectively (Figure 1). Additionally, the APACHE II was chosen by its easy retrospective application, the experience accumulated with its use in front of various clinical and surgical situations and of the capacity to serve as an indicator of changes in the attendance politics of that same Institution during distinct periods [13-19].

The Student's t-test was used to determine the statistical significance of the differences between means. For statistical analysis, the programs Microsoft Excel 2003 (Microsoft, Seattle, WA, USA) and Minitab, 13th version (Minitab Inc., State College, PA, USA) were used. For ROC curve analysis, the SPSS 13.0 for Windows (SPSS inc., Chicago, Illinois, USA) was used. For multiple logistic regression analysis, the SAS 9.0 (SAS Institute Inc, Cary, NC, USA) was used. The level of statistical significance was set at P < 0.05.

## Results

Regarding general demographic data (Table 1), overall mean age was significantly higher during the second period, mainly among patients with neurological afflictions. Age distribution was relatively uniform during the two study periods, except for the significant reduction in the number of patients from 20 to 29 years of age observed in May 2001. No significant differences in gender or origin were observed.

**Table 1:** Demographic characteristics of the study population

**Variables**	**May 1996**	**May 2001**	**P**	**95% CI**
1. Age				
Overall	49.0 ± 0.9	52.0 ± 0.9	0.02	(0.45; 5.69)
Internal Medicine	50.0 ± 1.3	52.0 ± 1.2	0.30	(-1.61; 5.18)
Surgery	47.0 ± 1.9	49.0 ± 2.2	0.41	(-3.47; 8.41)
Neurology	49.0 ± 1.8	57.0 ± 2.3	0.01	(1.89; 13.46)
2. Distribution by age bracket				
13 |- 20	19.0	21.0	0.57	(-0.04; 0.03)
20 |- 30	74.0	45.0	0.01	(0.01; 0.10)
30 |- 40	79.0	60.0	0.24	(-0.02; 0.07)
40 |- 50	78.0	72.0	0.73	(-0.05; 0.04)
50 |- 60	65.0	63.0	0.75	(-0.05; 0.03)
60 |- 70	67.0	68.0	0.45	(-0.06; 0.03)
70 |- 80	58.0	66.0	0.22	(-0.07; 0.01)
80 |- 90	28.0	28.0	0.88	(-0.03; 0.02)
90 |-	06.0	09.0	0.32	(-0.02; 0.01)
3. Gender				
Male	301.0	247.0	0.06	(0.00; 0.12)
Female	173.0	185.0	0.06	(0.00; 0.12)
4. Origin				
Ribeirão Preto (city)	351.0	298.0	0.10	(0.00; 0.10)
Ribeirão Preto (region)	123.0	134.0	0.10	(0.00; 0.10)
**TOTAL**	474.0	432.0	---	---

95% CI, 95% confidence interval

From 1996 to 2001, patient distribution among the three clinical areas studied demonstrated a proportionally higher demand for treatment within the area of internal medicine at the expense of neurology, whereas demand for surgery remained relatively stable. A significant increase in the overall mortality rate was also observed, this was actually due to the increase in mortality rate observed in patients admitted to the area of internal medicine. However, once adjusted by APACHE II mortality risk, using a multivariate logistic regression analysis, with the exception of the overall mortality, there was no differences between the two periods in the three clinical areas studied. In addition, there was an increase in the percentage of patients admitted with co-morbidities, especially within the areas of internal medicine and surgery, and an increase in the percentage of patients with one or more hospital complications during the hospital stay, again, within the areas of internal medicine and surgery (Table 2).

**Table 2:** Evolution of distributions according to specialty, percentage of complications, mean mortality rate and presence of co-morbidities

**Variables**	**May 1996**	**May 2001**	**P**	**95% CI**
*1. Clinical areas*				
Internal medicine	260.0	284.0	<0.01	(0.04; 0.17)
Surgery	107.0	79.0	0.11	(-0.01; 0.09)
Neurology	107.0	69.0	0.01	(0.01; 0.10)
2. Mortality rate observed (%)*				
Overall	6.1	12.2	<0.01	(0.08; 0.31)
Internal medicine	5.0	11.6	0.10	(-0.01; 0.17)
Surgery	5.6	8.8	0.41	(-0.07; 0.18)
Neurology	9.3	18.8	0.14	(-0.07; 0.47)
*3. Presence of co-morbidities (%)*				
Overall	66.6	77.0	<0.01	(0.04; 0.16)
Internal medicine	73.4	80.9	0.04	(0.01; 0.14)
Surgery	47.6	64.5	0.03	(0.02; 0.31)
Neurology	69.1	75.3	0.47	(-0.19; 0.07)
*4. Percentage of complications observed during the hospital stay*				
Overall	11.9	31.0	<0.01	(0.13; 0.24)
Internal medicine	13.4	31.3	<0.01	(0.10; 0.24)
Surgery	8.6	21.5	0.02	(0.02; 0.22)
Neurology	31.7	36.2	0.65	(-0.19; 0.09)
*TOTAL*	474.0	432.0	---	---

95% CI, 95% confidence interval

*Value of P and 95% CI are results expressed after adjustments done by the APACHE II score index, using the multiple logistic regression analysis.

The mortality risk based on the APACHE II score was higher among patients admitted in May 2001 than among those admitted in May 1996, although this result only attained statistical significance among patients admitted to the area of internal medicine (Table 3).

**Table 3:** APACHE II mortality risk in the study periods

**Variables**	**May 1996**	**May 2001**	**P**	**95% CI**
Overall (mean ± sd)	12.0 ± 0.5	14.8 ± 0.6	<0,01	(1.20; 4.40)
Internal medicine (mean ± sd)	11.5 ± 0.6	15.3 ± 0.8	<0,01	(1.80; 5.90)
Surgery (mean ± sd)	13.9 ± 1.1	14.7 ± 1.2	0.60	(-2.40; 4.10)
Neurology (mean ± sd)	11.5 ± 0.6	15.3 ± 0.8	0.52	(-2.80; 5.60)

95% CI, 95% confidence interval

## Discussion

The MRO was implemented to manage patient flow between the network of health services, thus matching patient need for care with the service complexity and capacity of the respective hospital units. The impact on the health care indicators for the EC was immediate, and those indicators have fluctuated little over the recent years. Between the years 1996 and 2001 the number of consultations was reduced from 139,137 to 50,214 and admissions fell from 19,511 to 11,927, while the occupancy rates fell from 104.6% to 88.4% [4]. The observed data, in association with the increase in mortality risk based on the APACHE II score, demonstrate the technical and management capacity of the MRO in ordering healthcare access within the University Hospital, as well as in directing the less serious afflictions to other centers of the emergency network

Initially, health managers believed that the MRO and SAMU services would bring about a reduction in emergency-room admissions of low severity cases, but that the severity profile of the patients admitted would not be affected. However, after the operation of MRO, the health care teams had the perception that the hospital had begun to receive a larger number of elderly patients with higher incidences of co-morbidity and, consequently, higher incidences of complications during the hospital stay and higher mortality risk, mainly in the Internal Medicine Department. This study has confirmed the perception of health care teams and has shown that the joint work of MRO and SAMU, in addition to redirecting the low severity cases for lower complexity emergency care centers, extended the access of critical care to patients from the reference centers, contributing to the consolidation of the principle of equity.

The evolution observed in the pattern of hospitalization and in the severity profiles mirrors the changing demographic and epidemiologic profiles of populations in other developed and developing regions, including Brazil. However, the relative age increase of about three years of the patients admitted in May of 2001, when compared with the admitted ones in May of 1996, placed patients above the average age of the Brazilian population between these two periods by about eight months [6].

It is important to emphasize that, beyond the role of the SAMU and MRO in organizing access to the network of emergency services, the perceived and confirmed results induced the managers of the university hospital, as well as those responsible for the municipal and regional public health system and for the University, to adopt new strategies for teaching and practicing emergency care.

These results induced the health managers of the EC to reorient the internal spaces. Among the 16 beds of the emergency room, 5 were transformed into beds of clinical stabilization. Among the 108 beds assigned to the ward of internal medicine, neurology and surgery, 10 have been transformed into a unit of intermediate care and 10 into an isolation unit for contagious diseases. There was an increase in the number of intensive care beds for adult patients (from 7 to 16). The University of São Paulo School of Medicine at Ribeirão Preto, despite a good deal of resistance on the part of its directors, has increasingly shifted its academic activities, and even its research activities, from the University Hospital to the network of primary and secondary care facilities [4]. The managers of the public health system and the academy are learning that it is no longer possible to provide all levels of health care at a tertiary-care hospital.

## Conclusion

On the basis of the data analyzed and the evaluation system adopted, we conclude that the medical regulation of the flow of patients and the control of the dispatch of mobile resources have contributed to the increased severity profile of patients presenting with non-traumatic emergencies to the Internal Medicine Department of the Emergency Care Unit. This process, despite the conflict observed between professionals, managers of the public health system and the academy, gained popular support and induced the internal reorganization of the hospital with an increase in the number of intensive care beds, and promoted the replacement of didactic and medical assistance activities in a non-hospital unit for emergency care, forcing the primary and secondary levels of care to assist in low complexity emergencies, referring only the more complex ones to the tertiary level of care.

## Competing interests

The author(s) declare that they have no competing interests.

## Authors' contributions

SLBL conceived the study and designed the trial, undertook the data collection, participated in the statistical analyses, drafted the manuscript and takes responsibility for the paper as a whole. JSS participated in conceiving the study, supervised the conduct of the trial and data collection and provided statistical advice on study design. SS helped in supervising the conduct of the trial and data collection and in providing statistical advice on study design. All authors read and approved the final manuscript.

## Pre-publication history

The pre-publication history for this paper can be accessed here:


